# Contagion Vivum

**Published:** 1890-10

**Authors:** 


### Contagion Vivum.

The indiscriminate use of dental instruments is doing much to spread the actinomycosis among our people. This parasite presents many features in common with tubercular growth; they are commonly found in the mouth, tonsils, parotid, buccal glands, and in the form of nodules upon the jaw; pass down to the lungs and liver, giving rise to grave pathological changes. The organism causing these lesions belongs to the order of algae; distinct from the tubercular bacilli. It is allied to the hypomycetes, similar to the aspergillus, or pebrine of silkworms.

It is most infectious; once it gets on the forceps or filling instruments of a dentist it is just as difficult to root it out of his office as to wipe out the spores of the tinea sycosis from a barber shop.

It is a latent, unsuspected source of lung and bronchial trouble among all who patronize a dental office.

Sooner or later the State must take recognition of this source of a terrible malady which is difficult to kill or sterilize. A free use of the anti-microbe powder, as a mouth wash, thrice daily, forever eradicates this germ from the oral cavity. Either that, or the chemically pure peroxide of hydrogen should be the leading feature in all dental offices.—From *The Germicide*.

